# Assessment of pixel-oriented k-NN machine learning algorithm performance for the interannual remote sensing monitoring of eelgrass beds at the mouth of the Romaine

**DOI:** 10.1007/s10661-023-11468-3

**Published:** 2023-07-12

**Authors:** P. Lemieux, C. Lalumière, N. Fugaru, J.-P. Gilbert, A. Tremblay

**Affiliations:** 1 Environmental Studies & Climate Changes, Englobe Corp, Englobe 1001, Rue Sherbrooke Est, Bureau 600, Montréal, QC H2L 1L3 Canada; 2 Environmental Studies & Climate Change, Englobe Corp, Boul. du Parc-Technologique, Bureau 200, Englobe, Québec, QC 505H2L 1L3 Canada; 3 Innovations, Groupe Alphard, 1255, Boul. Lebourgneuf, Bureau 480, Québec, QC Canada; 4 Direction Environnement, Hydro-Québec, Place-Dupuis, 800 de Maisonneuve, 23E Étage, Montréal, QC Canada; 5 Direction Environnement, Hydro-Québec, Place-Dupuis, 800, De MaisonneuvePlace-Dupuis, 800 de Maisonneuve, 23E Étage, Montréal, QC Canada

**Keywords:** Eelgrass, K-NN, Classification, Machine learning, Pixel oriented

## Abstract

Eelgrass cover extent is among the most reliable indicators for measuring changes in coastal ecosystems. Eelgrass has colonized the mouth of the Romaine River and has become a part of environmental monitoring there since 2013. The presence of eelgrass in this area is an essential factor for the early detection of changes in the Romaine coastal ecosystem. This will act as a trigger for an appropriate environmental response to preserve ecosystem health. In this paper, a cost- and time-efficient workflow for such spatial monitoring is proposed using a pixel-oriented k-NN algorithm. It can then be applied to multiple modellers to efficiently map the eelgrass cover. Training data were collected to define key variables for segmentation and k-NN classification, providing greater edge detection for the presence of eelgrass. The study highlights that remote sensing and training data must be acquired under similar conditions, replicating methodologies for collecting data on the ground. Similar approaches must be used for the zonal statistic requirements of the monitoring area. This will allow a more accurate and reliable assessment of eelgrass beds over time. An overall accuracy of over 90% was achieved for eelgrass detection for each year of monitoring.

## Introduction

One of the main goals of environmental follow-up is to assess the actual environmental impacts of development projects with a view to conserving and protecting biodiversity. A case in point is eelgrass (*Zostera marina L.*), an aquatic plant that plays a key role in maintaining the structure and the function of coastal ecosystems (DFO, 2009; Duarte & Chiscano, 1999; Hemminga & Duarte, 2000; Hily & Bouteille, 1999; Vandermeulen, 2009). The vulnerability of eelgrass to anthropogenic disturbances and the speed with which it responds to various stressors (e.g. sedimentation, turbidity, nutrients, currents and scour) (Vandermeulen, 2005; Vandermeulen et al., 2012) make it a preferred indicator species for the follow-up of environmental impacts.

In terms of both area and spatial distribution, the extent of eelgrass beds is one of the most accessible features that may be used to identify and assess interannual changes (Schweizer et al., 2005). For several decades now, remote sensing has been used to map coastal habitats (CIDCO, 2006; Provencher & Deslandes, 2012). While photointerpretation was initially preferred (DFO, 2009), remote sensing quickly established itself as a more cost-effective alternative based on objective optical detection criteria, unlike photointerpretation, which is more subjective (CIDCO, 2006; Provencher & Deslandes, 2012).

Already widely used, satellite imaging provides good quality spectral inputs for varied environmental monitoring of bio-marine instances, such as the presence of different algae beds. By leveraging the discrimination power of spectral bands, the presence of eelgrass has usually been detected through vector object (e.g. polygon) segmentation and classification. For several years now, segmentation and classification based on the spectral approach have been recognized by the scientific community and widely used (Hossain et al., 2015). The various satellite imagery-based detection and mapping techniques in use require classification methods involving supervised or unsupervised machine learning techniques. Field data collection methods, as well as the types of data collected and object-oriented processing and classification techniques, have contributed over time to the standardization of these remote sensing methods, which are now commonly used in the field of geomatics.

Advances in computer hardware and the availability of high-resolution data associated with new classification tools have led to improvements in the detection, segmentation, classification and mapping of indicators, such as intertidal aquatic beds, and have allowed for the continuous improvement of established approaches with a view to streamlining the follow-up of environmental impacts. In the last few years, the pixel-oriented approach, based on raster high-resolution data, texture resemblance criteria and the pixel neighbourhood for edge detection, has become the subject of machine learning classification development. This new pixel approach algorithm, applied on orthorectified panchromatic satellite imaging, provides high-quality raster inputs (Saifi et al., 2020) and can stand as a potential alternative cost- and time-effective remote sensing method that covers large and inaccessible areas nearly instantly and frequently. The study area located at the mouth of the Romaine River has already been subject to eelgrass monitoring by classic and costly on-field detection methods and the fieldwork conducted has shown insignificant presence of other algae families. The present study focuses on efficiently detecting eelgrass beds and their spatial occupancy, setting aside the question of eelgrass subfamily segregation.

It is in this context that, during the interannual follow-up monitoring of eelgrass beds at the mouth of the Romaine, the pixel-oriented approach was integrated into machine learning-based classification using the k-nearest neighbours (“k-NN”) algorithm (Effrosynidis et al., 2018).

The main goal of this study is to evaluate and assess the performance of a workflow focused on the pixel-oriented k-NN algorithm and applied to various processing modellers with the aim of efficiently mapping the interannual eelgrass covering. Due to its reliance on similarity, texture and pixel neighbourhoods’ criteria, this classification method has demonstrated computational robustness and efficiency (OpenCV, 2015) enabling its use with satellite imagery. Another goal of the exercise is to explore whether the k-NN algorithm and discrimination analysis are capable of efficient binary classification for the coastal study area, and to determine whether the detection accuracy will change along with the field observation collection method (i.e. either set of close transect observations or sets of distant observations randomly distributed in the monitoring area). Based on the ability of the k-NN OpenCV library to cope with errors during the execution process of the classification and final results obtained, it is useful to specify that the computational robustness of this approach allows its application to other environmental follow-up exercises that require remote sensing. Given the number of follow-up campaigns carried out since 2013 (four), it has been possible to assess the effect of the two field-surveying methods (either transect observation or distant random observations) on the results.

## Follow-up area

The Romaine project involves the construction of a 1500-MW hydroelectric complex on the Romaine River in the Côte-Nord region of Québec. It includes four generating stations and four reservoirs having a total area of 279 km^2^. The Romaine complex will have a regulated freshwater discharge and an average annual energy output of about 8 TWh.

Local communities were concerned that the change in freshwater flow could influence the ecosystems at the mouth of the Romaine River.

**Fig. 1:**
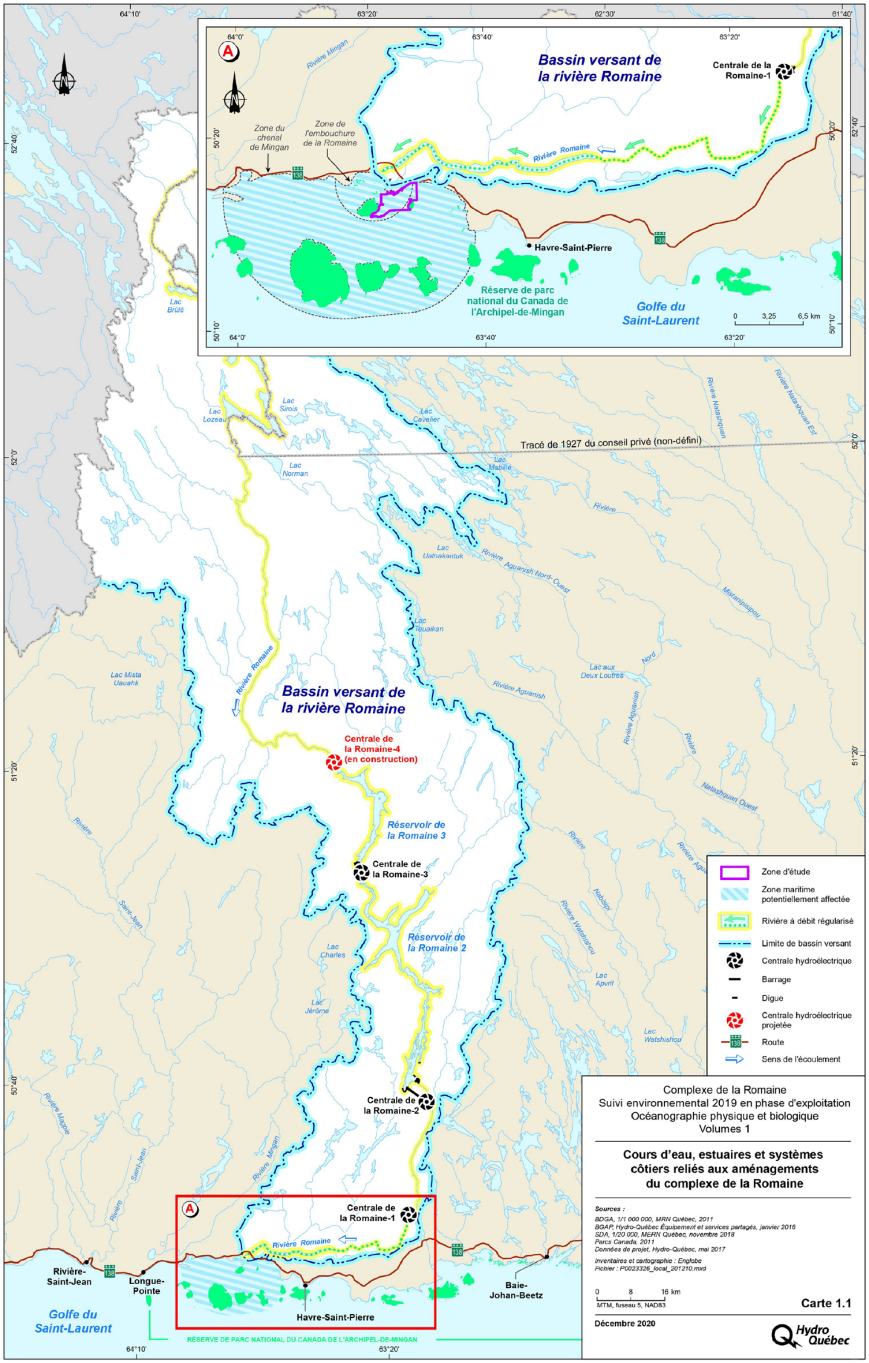
Eelgrass monitoring area at the mouth of the Romaine

Eelgrass beds provide a habitat sought by several animal species (e.g. Mysidacea, threespine stickleback, fourspine stickleback, blue mussel and duck) to fulfil some of their biological functions and play a role in marine biodiversity. Eelgrass beds are present in the lower intertidal habitats at the mouth of the Romaine (Hydro-Québec, 2007) and these eelgrass beds are concentrated between La Grosse Romaine and La Petite Romaine islands, as well as along the coast east of Pointe à Aisley. It is divided into two distinct sectors, i.e. the Eastern and the Western sectors (Fig. 1).

Taking into account the eelgrass height responsiveness to changes in water quality (Fonseca et al., 1992; Duarte and Chiscano, 1999) and the concerns of local communities, the eelgrass presence was integrated into the Romaine complex environmental follow-up program in 2013. The presence or absence of eelgrass in the study area is a key indicator for the early detection of environmental changes in the Romaine coastal ecosystem and will act as a trigger for an appropriate environmental response to preserve the ecosystem health.

To this end, remote sensing techniques using available high-resolution panchromatic satellite products were selected as the preferred monitoring procedure to track the interannual evolution of the extent eelgrass presence on the study area. To date, monitoring activities have been conducted for four years: 2013, 2015, 2017 and 2019. Many other environmental parameters are also measured, such as water temperature and salinity, to follow changes in eelgrass habitats.

## Methodology

### Selection of reference satellite images

**Fig. 2:**
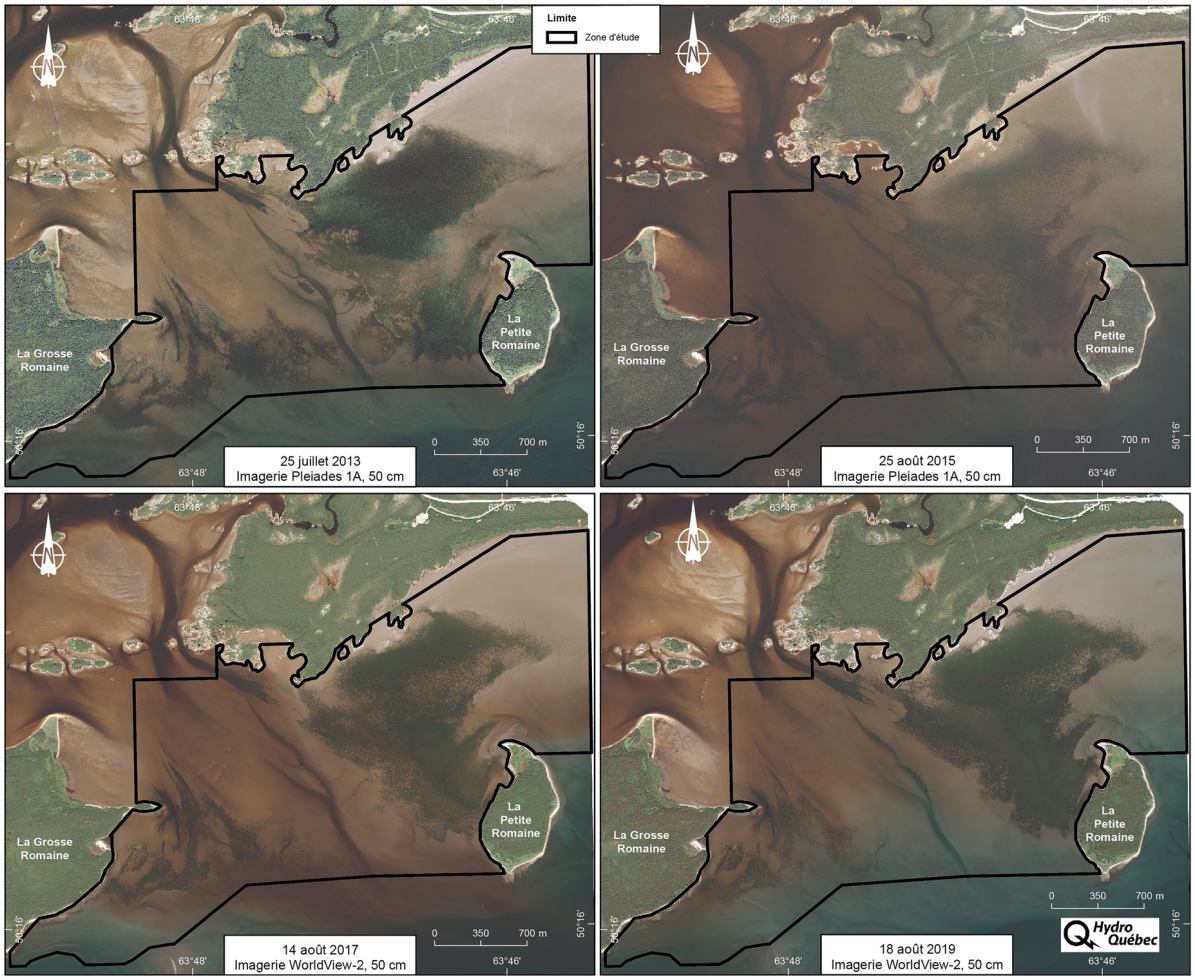
Satellite images acquired for each follow-up year

For each follow-up year (2013, 2015, 2017 and 2019), a set of satellite images was acquired to cover the monitoring area at the mouth of the Romaine (Table 1). Depending on the year, preprocessed panchromatic pansharpened images were acquired from a long-stand satellite image provider (Effigis, 2021) using either the Pleiades-1A satellite (2013 and 2015) or the WorldView-2 satellite (2017 and 2019) (Fig. 2). In addition to obtaining full coverage of the monitoring area, it was also important to achieve the following conditions to allow for an optimal detection of eelgrass: high resolution (50 cm), satellite as close to the nadir point as possible (less than 20°), optimal eelgrass bed growing period (generally August), lowest tide and minimal cloud cover and wave effects (less than 3%). Satellite image acquisition should coincide with field operations as much as possible.

**Table 1:** Characteristics of satellite imagery acquired in 2013, 2015, 2017 and 2019

Follow-up year	Date	Hour	Satellite	Georeferencing accuracy (m)	Resolution	Raster bands
2013	July 25	14:47	Pleiades-1A	0.86	50 cm	3(RGB)^1^
2015	August 25	14:52	Pleiades-1A	0.15	50 cm	3(RGB)^1^
2017	August 14	15:20	WorldView-2	0.60	50 cm	3(RGB)^1^
2019	August 18	15:24	WorldView-2	0.24	50 cm	3(RGB)^1^

^1^*RGB* red, green and blue

Once acquired, the 8-bit Pleiades-1A and WorldView-2 images first underwent a basic pan-sharpening process, which included filtering to reduce imperfections, colour enhancement and contrast adjustment to highlight eelgrass beds. The images were then projected into the NAD83 (CSRS)/MTM zone 5 reference frame and then clipped to yield sub-images of the monitoring area.

### Field observation points

**Fig. 3:**
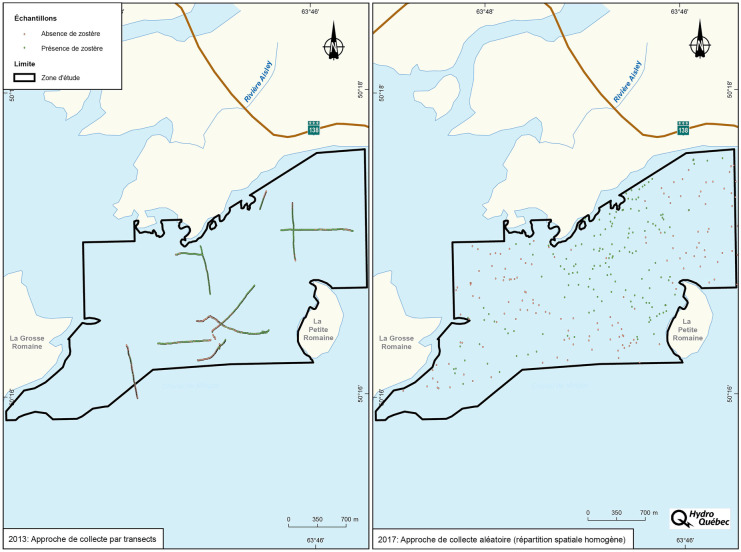
Field data collection by transect surveying (2013) and random surveying (2017)

To calibrate and validate the satellite image processing model, data on the presence or absence of eelgrass was collected in the field during each follow-up year (Table 2). In assessing the performance of the classification approach, two surveying methods were used to collect data on the presence or absence of eelgrass. In 2013 and 2015, high-density observation data was collected along transects, while, in 2017 and 2019, data was collected at random locations in the monitoring area (Fig. 3). The random surveying method was found to be more suitable as it enhanced the accuracy of the model through improvements in the factors below:

- Distribution of training and validation data within the monitoring area
- Delimitation of eelgrass bed boundaries
- Eelgrass presence/absence ratio

**Table 2:** Number of eelgrass presence/absence observations at the mouth of the Romaine for each follow-up year

Follow-up year	Date	Number of eelgrass presence observations	Number of eelgrass absence observations	Total number of observations	Proportion of eelgrass presence observations (%)	Surveying method
2013	Aug. 11–13 Aug. 15–16	831	562	1,393	59.7	Transect
2015	Aug. 26 Sept. 2	687	578	1,265	54.3	Transect
2017	Aug. 20	149	154	303	49.2	Random in the monitoring area
2019	Aug. 20 and 23	73	48	121	60.3	Random in the monitoring area

**Fig. 4:**
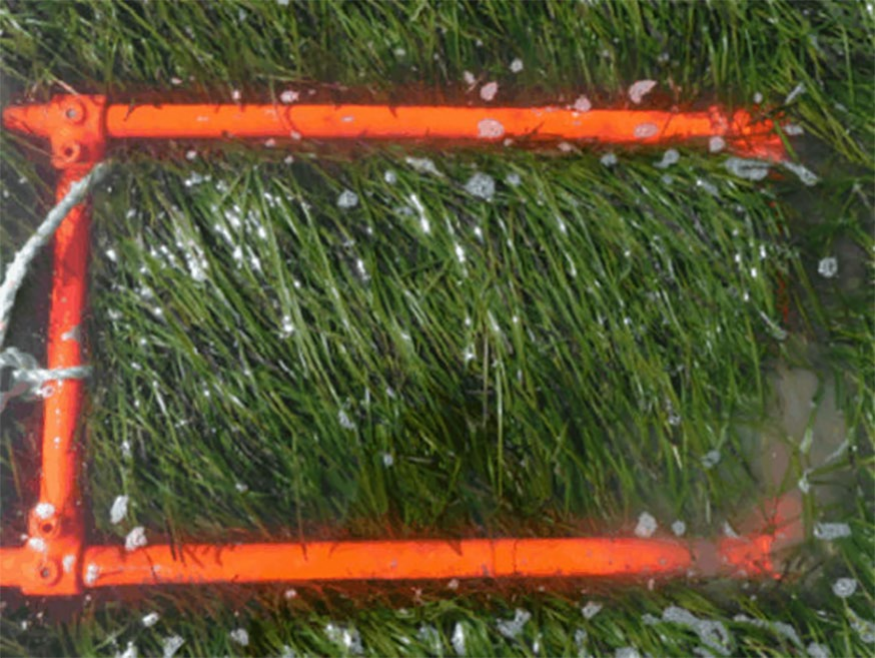
Example of quadrat used

Field observations of the presence or absence of eelgrass were made during the second half of August in each follow-up year. This period was preferred because it is the time of the year when eelgrass growth reaches its maximum. Data was collected directly in the field through observations made on foot or by boat to cover the extent of the eelgrass bed in deep waters. In both cases, a 30 cm × 50 cm quadrat was placed on the ground (Fig. 4). The presence or absence of eelgrass inside the quadrat was noted. In addition, a GPS point was acquired using an RTK device.

### Imagery data preparation

First, the processing area was defined on water depth: 0 m to approximately 6 m based on the coastal outline found in Hydro-Québec’s map library and the interpretation of heterogeneous bathymetric data provided by the Canadian Hydrographic Service (depth below 5.5–6.0 m depending on the resolution). This bathymetric data-based delimitation is consistent with the high minimum light requirement of eelgrass (DFO, 2009) and its general tendency to grow in shallow waters in Québec. The limits of this shallow-water area were edited by superimposing them over satellite images to ensure no potential eelgrass bed was excluded from the analysis. The image was then masked off using the edited shallow-water area using ArcGIS v10.5.1 to improve the image aspect in the monitoring area.

To optimize edge detection, a natural and infrared component enhancement of the orthoimage was performed by the satellite image provider using Photoshop. In doing so, image pixels were filtered to avoid missing data in the monitoring area; then, the images were compressed into lossless JPEG 2000 format (using Global Mapper) to achieve a fully transparent background with no colour gradation. The substrate hue—marking the absence of eelgrass—is water depth dependent, which can impair eelgrass detection. The matter was addressed by using the red and green band raster PCA transformation (Demšar et al., 2013). New PCA rasters reduced the outliers generated by the substrate hue (water depth effect) and consequently enhanced the feature detection.

For WorldView-2 and Pleiades-1A satellite imagery data, the maximum average orthorectification error was found to be ± 0.60 m and ± 0.86 m, respectively, for X (East) and Y (North) coordinates. For field observations, the average measurement error was found to be ± 0.05 m. These errors result in an offset between eelgrass presence data collected in the field and its footprint in the imagery. The red, green and blue bands were therefore resampled to 1 m per pixel in the monitoring area to ensure field observations were included in the same pixel that was used for classification.

RGB raster bands were first segmented into homogeneous polygon objects using the region growing segmentation algorithm to allow for the automatic detection (generation) of raster control points. To this end, the SAGA GIS Free Open Source Software (SAGA GIS, 2015) was used to generate polygon objects with homogeneous ranges starting from seeded groups of pixels that met similarity and neighbourhood criteria. The automatic generation parameter acts as a detection threshold for grouping neighbouring pixels with close values. If the value difference for neighbouring pixels in a region of the image is less than 1, all those pixels are deemed to belong to the same grouping and a segmentation control point is entered. From that point on, the segmentation algorithm is applied (Adams & Bischof, 1994). The generalization index chosen for the SAGA algorithm (i.e. 1) is equivalent to the similarity criterion; it represents the grouping threshold for nearest neighbour pixel values (Bechtel et al., 2008). These two parameters allow for a reasonably accurate delimitation of polygons regarding the geometric orientation of the objects.

Lastly, classification inputs (predictor and target variables) were prepared. Predictor variables correspond to the statistical features of polygon objects obtained using the QGIS zonal statistics tool (average, median, minimum, maximum, neighbouring distance and neighbouring index). Target variables correspond to the presence or absence of eelgrass in the monitoring area. To this end, the randomly collected field data was divided into two categories, i.e. data used to create training polygons and data used for validation, thus matching the categorization used to create the monitoring baseline in 2013. Validation data was only used to determine the accuracy of the final prediction, which was prepared using training polygons. Training data were assigned to polygon objects.

### K-nearest neighbour (k-NN) classification method

Since the density of eelgrass beds is heterogeneous and their geographic distribution is uneven along the coast, pixel-oriented k-nearest neighbour (k-NN) classification was used to determine whether the plant is present or absent. This method is commonly used for feature extraction using artificial intelligence techniques, especially automated learning (Alpaydin, 2010). In the context of the follow-up monitoring of eelgrass beds at the mouth of the Romaine, this method was chosen to classify preprocessed scenes. Specifically, the pixel-oriented k-NN method consisted in using the eelgrass presence/absence value associated with various training polygon objects to generate a model that could be used to predict the presence or absence of eelgrass in the raster images for which no field data was collected (Franco-Lopez et al., 2001).

To create a training set for the k-NN algorithm, field observations were associated with the polygon objects generated through segmentation. After a simple field data overlaying procedure, if several points fell within the same polygon, a majority voting procedure was used to decide which eelgrass presence/absence value was assigned to the polygon. If a vote resulted in a draw (50/50), the polygon was excluded from the training set. To achieve better training data distribution and classification, polygon objects for which the absence of eelgrass was obvious were added by an eelgrass expert (using photointerpretation). In addition, photointerpretation helped distribute observations over the entire monitoring area to represent the full range of image hues. At least two classification iterations using the k-NN method were necessary to determine photointerpretation needs, especially at model boundaries. To maintain the detection power of the k-NN algorithm, photointerpretation was used only to identify locations where the absence of eelgrass was obvious while allowing the algorithm to decide whether the plant was present in raster images.

Meta-parameters were selected based on the statistical characteristics of the monitoring area to avoid an excessive number of trials. The model was built using SAGA GIS (O. Conrad © 2016) based on the OpenCV machine learning concept (Bishop, 2006). The algorithm takes all training set samples (or polygon objects) and predicts the response for a new pixel by analyzing a given number (k) of the points nearest to the sample through a weighted sum formula (Franco-Lopez et al., 2001). This method is called “learning by example” as it seeks to identify the vector with a known response that is the closest to the vector given to establish the prediction. This approach is widely used in many fields due to its simplicity, theoretical clarity and the excellent performance of the classification thus obtained. The approach used for eelgrass monitoring consists in applying the method directly to the processed satellite images. The training data stemmed from field observations.

The k-NN classification algorithm allows for the detection, recognition and classification of the features of an object in a raster image based on its class (Gollapudi, 2019). The nearest neighbour (NN) method consists in detecting unknown objects of a given class in an image based on their nearest neighbour since the unknown classes make up the training classes. The algorithm is based on the NN rule, where the nearest neighbour is calculated using the k-value to specify the number of nearest neighbours to take into consideration when defining a class of sample data points (Wang, 2019).

**Fig. 5:**
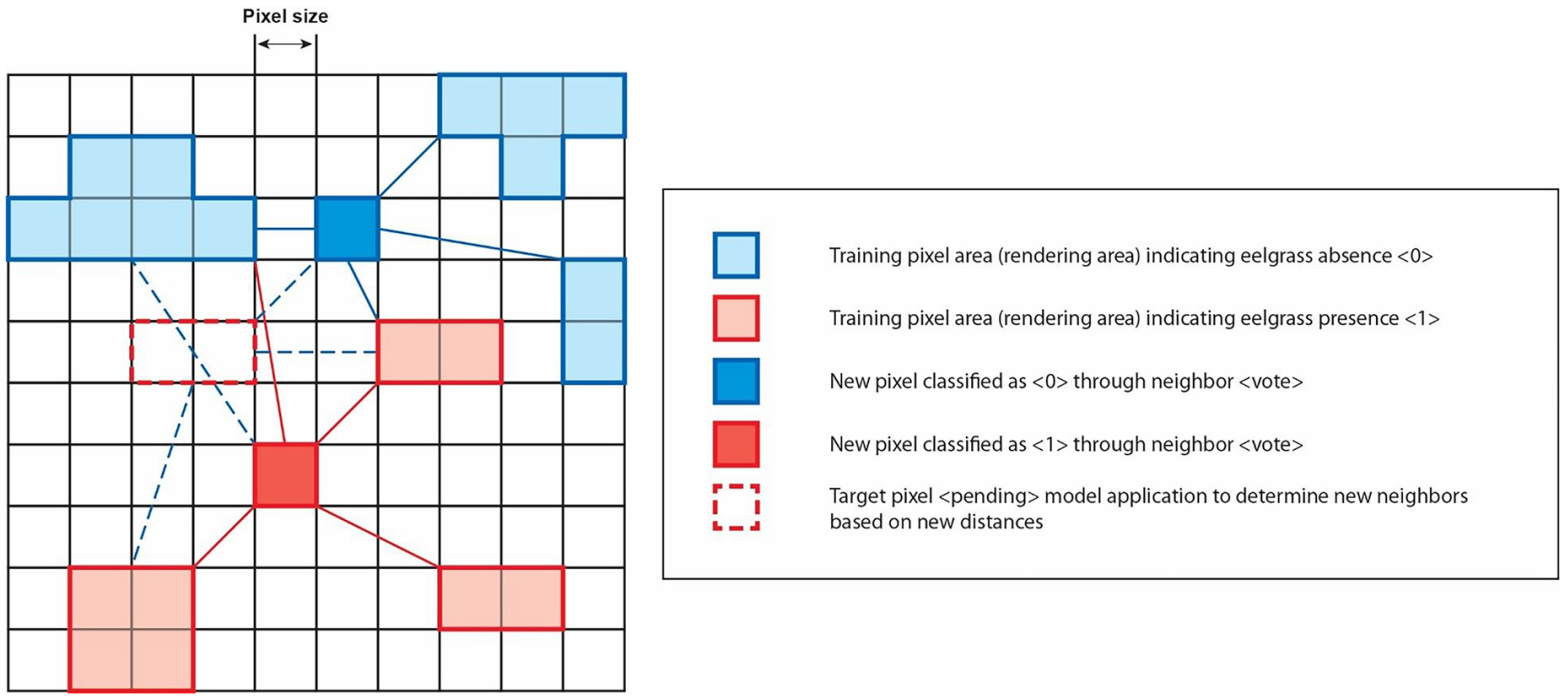
Pixel-oriented k-NN classification algorithm implementation diagram

This study relied on the SAGA free open-source software (SAGA GIS 2015), which incorporates the OpenCV machine learning library including the k-NN raster classification algorithm. When used for pixel-oriented classification, the k-NN algorithm assigns to each unidentified (target) pixel the features of the reference pixels (training set of pixels) for which field data is available that most closely match it (Franco-Lopez et al., 2001). The similarity criterion is defined in a characteristic space, i.e. the Euclidean or Mahalanobis distance. For this study, the Euclidean distance function was used (see Eq. (1)) to measure the distance between pixels to be classified and the reference pixel fields (Fig. 5). Therefore, to classify a new (target) pixel, its distance from each model pixel was calculated using the Euclidean distance formula (Fig. 5, Table 3). The distance from the most similar pixels—the nearest neighbours—was calculated and the new pixel was classified in the category that contained the greatest number of nearest neighbours. Consequently, the pixel-oriented k-NN method has the benefit of using the interpretation of all raster image pixels (automated learning) rather than relying on the classification of polygon objects to determine grouping values.

**Table 3:** General image training statistics for each follow-up year

Follow-up year	Nearest neighbour Index (NNI)	Average nearest neighbour distance (m)	Average number of nearest neighbours
2013	1.15	3.5	4.0
2015	1.14	3.4	3.9
2017	1.13	3.7	4.1
2019	1.05	3.7	3.9

1 $${d}_{E }\left(x, y\right)= \sum\limits _{i=1}^{n}\sqrt{{{x}_{i}}^{2}+{{y}_{i}}^{2}}$$

### Validation of classification algorithm

The performance of the final model classification was calculated using validation polygon objects (Ratner, 2017). Accuracy was selected as the main performance indicator and the kappa coefficient was used as a complementary indicator. The kappa coefficient is a measure of the intensity of the agreement between paired qualitative judgments relative to the effect of random agreement.

To assess the performance of the classification algorithm, a confusion matrix (polygons/grid) was calculated using validation data (Table 4). The comparison between the resulting model and the validation polygons was characterized in terms of accuracy

- and coefficient of agreement (kappa). The value of kappa is centred around 0, with a negative value indicating disagreement, while a value of 0.6 signals strong agreement among observed values and the values predicted by the model, and a value of 1 indicates full agreement (Landis & Kock, 1977).

**Table 4:** Proportion (%) of in situ data used for training and validation

Follow-up year	Proportion (%) of in situ data used for training	Proportion (%) of in situ data used for validation
2013	66.9	33.1
2015	66.4	30.6
2017	63.7	36.3
2019	62.8	37.2

## Results

The performance of the final model classification was calculated using validation polygon objects, which were generated using the field observation data points. The overall accuracy was selected as the main performance indicator and the kappa coefficient was used a complementary one. Depending on the follow-up year, the accuracy of the eelgrass prediction model ranged from 90.6 to 98.1%, while the kappa coefficient ranged from 0.81 to 0.97 (Table 5). Based on the kappa values obtained, there is strong agreement between the observed values and the values predicted by the model for each of the follow-up years. The accuracy of the prediction model is due in large part to the classification algorithm used (pixel-oriented k-NN approach) since it allows for an optimal use of the raster information contained in the image (Immitzer et al., 2012) and hence its discrimination power. The fraction of incorrect predictions helps explain the accuracy of the model for all follow-up years. It was in the order of 10% of cases in 2013, 2015 and 2019, and 4.3% in 2017 (Table 5).

**Table 5:** Assessment of model performance using field observations (validation points) for each follow-up year

Follow-up year	Observation: absence	Observation: presence	Total	Overall accuracy (%)	Kappa
2013					
Prediction: no eelgrass	60	0	60	90.9	0.82
Prediction: eelgrass present	0	116	116		
Total	60	116	176		
No. of incorrect model predictions	7	9	16		
No. of correct model predictions	53	107	160		
2015					
Prediction: no eelgrass	79	0	79	90.4	0.81
Prediction: eelgrass present	0	78	78		
Total	79	78	157		
No. of incorrect model predictions	12	3	15		
No. of correct model predictions	67	75	142		
2017					
Prediction: no eelgrass	63	0	63	95.8	0.97
Prediction: eelgrass present	0	57	57		
Total	63	57	120		
No. of incorrect model predictions	5	0	5		
No. of correct model predictions	58	57	115		
2019					
Prediction: no eelgrass	16	0	16	90.2	0.91
Prediction: eelgrass present	0	25	25		
Total	16	25	41		
No. of incorrect model predictions	0	4	4		
No. of correct model predictions	16	21	37		

The factors that affect the performance of the pixel-oriented k-NN classification algorithm include the number and size of the polygon objects used for classification (Table 6). The results indicate that these two factors may be tied to the quality of the imagery acquired (Fig. 2) since the performance of polygon object-based segmentation performance depends on the dominance of the spectral data under analysis and highlight-related disturbances (Wesolkowski & Fieguth, 2001), something that is also reflected in the kappa coefficient values for follow-up years 2013 and 2015 (Table 4). In addition, the polygon size reduction achieved through the use of pixel-oriented k-NN classification rather than object-oriented classification improves the detection of the presence or absence of eelgrass in the more fragmented areas of eelgrass beds, especially at the boundaries (Table 6).

**Table 6:** Number and average size of polygon objects classified and segmented in 2013, 2015, 2017 and 2019 at the mouth of the Romaine monitoring area

Type of polygon	2013		2015		2017		2019	
	No. of polygon objects	Average polygon object area (m^2^)	No. of polygon objects	Average polygon object area (m^2^)	No. of polygon objects	Average polygon object area (m^2^)	No. of polygon objects	Average polygon object area (m^2^)
Classified	214,998	2.7	329,367	2.8	820,765	2.1	127,793	3.8
Segmented	22,370	26.4	34,921	26.2	66,460	25.8	18,615	26.0

## Discussion

The results show that the field data collection method is a key factor in the performance of the pixel-oriented k-NN classification algorithm. In this regard, modifications made to the field data collection (i.e. random surveying [2017 and 2019] versus transect surveying [2013 and 2015] and collection of data for eelgrass presence and absence in similar proportions) contributed to a significant improvement in the quality of eelgrass bed detection since the performance of the model is partly influenced by the spatial distribution of the data over the monitoring area (Fig. 2). The random collection of observations of the presence and absence of eelgrass is preferable to the transect method, given that the transect method yields a dense set of observations covering a limited part of the monitoring area, which does not allow for a full assessment of the diversity of spectral band (RGB) hues associated with the presence or absence of eelgrass in the monitoring area. Conversely, the availability of a relatively similar number of field observations covering the entire monitoring area helps improve the performance of the model. The availability of georeferenced data on eelgrass presence/absence helps improve the definition of eelgrass bed boundaries, which are often less dense and somewhat fragmented.

To streamline the remote sensing of eelgrass beds, special care must be taken to synchronize field operations with the acquisition of satellite images. Indeed, the more dynamic the phenomena being observed or measured in the field, the closer in time the image data and the field work must be (McCoy, 2005). Since 2013, imagery segmentation results have shown that there is no significant interannual variation of the RGB footprint. Related to the average distance between nearest neighbours for a hypothetical random distribution, the nearest neighbour index (NNI) has tended to decrease, going from 1.15 (2013) to 1.05 (2019) (Table 3). The NNI tends to approach value of 1, thus showing a trend towards clustering. When this indicator is greater than 1, the trend is always towards dispersion or competition. The eelgrass bed maps for 2013 and 2015 are quantitatively and qualitatively comparable and the results show that the k-NN algorithm implementation methodology and its pixel-oriented application are robust. The eelgrass bed maps for 2013 and 2015 may be classified as very reliable (Landis & Kock, 1977). However, results can vary, even when using similar data sources. Therefore, to provide consistent monitoring over the long term, a repeatable, user-independent or semi-automated approach is recommended to produce comparable results over time (Shapiro et al., 2015). In this case, the performance of the pixel-oriented k-NN algorithm has been above average due to the use of a reproducible approach to image preprocessing, including a classification preparation procedure based on nearest neighbour statistics. Results for 2017 and 2019 show an excellent classification performance since the kappa index exceeds 0.9, which is an exemplary measure of agreement according to the scale proposed by Landis and Kock (1977). The model accuracy is significantly greater than for 2013 and 2015 (Table 5).

**Fig. 6:**
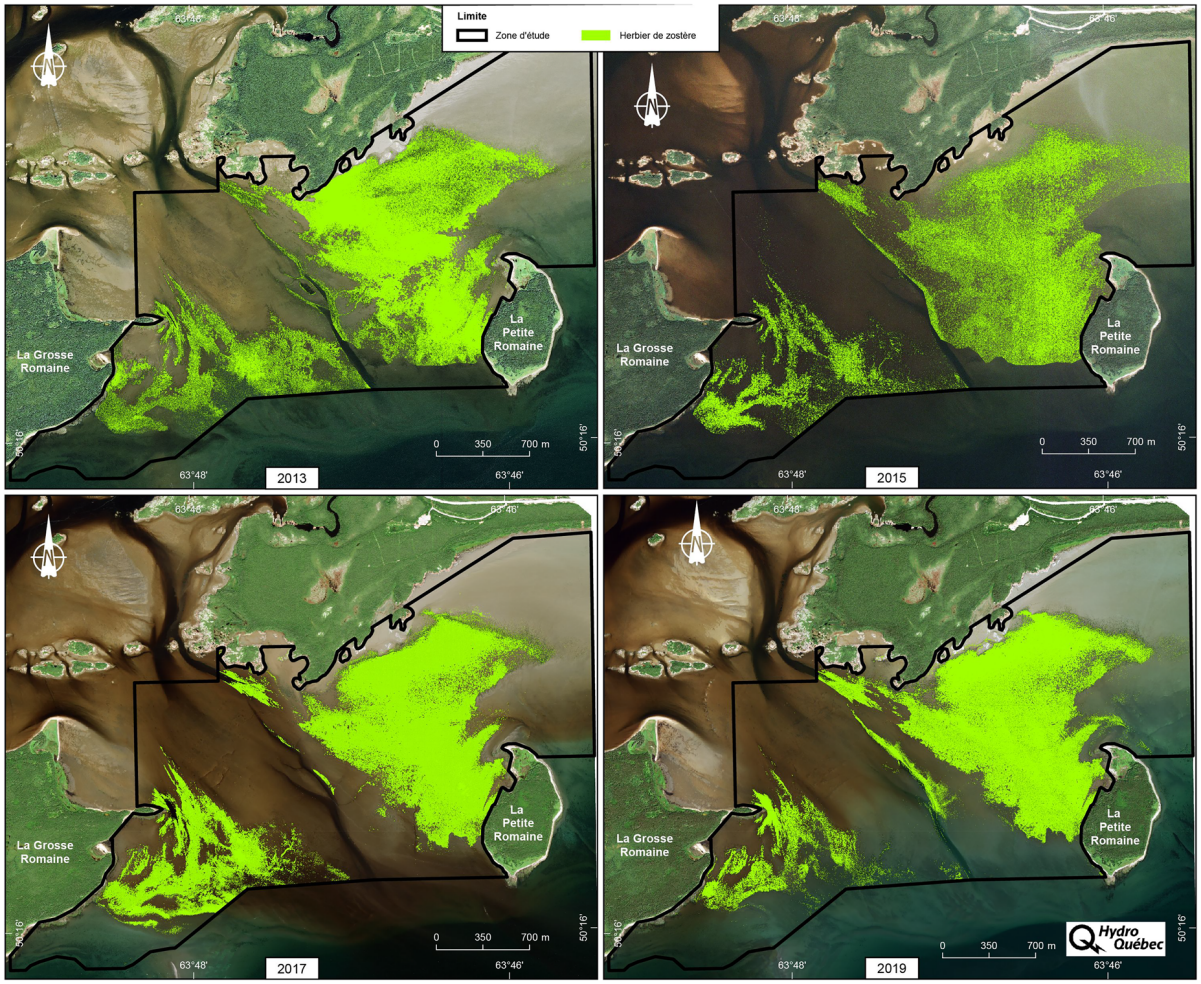
Map of eelgrass bed extent in monitoring area for 2013, 2015, 2017 and 2019

Lastly, using the classified images, it was possible to calculate the extent of the eelgrass bed and the coverage monitoring indicators. The area of eelgrass beds at the mouth of the Romaine is the most direct indicator that can be used to assess the condition of the eelgrass beds on each follow-up year and to perform interannual comparisons. This area was calculated using the vectorized results of the classification performed using the pixel-oriented k-NN method (Fig. 6).

## Conclusions

Based on the results obtained for the follow-up of eelgrass beds at the mouth of the Romaine between 2013 and 2019, it has been shown that the use of the pixel-oriented k-NN algorithm offers significant benefits for the environmental monitoring of components for which the main indicator is their extent or geographical distribution. Pixel-oriented k-NN classification is a high-performance algorithm with great computational robustness, which streamlines the assessment of interannual changes in beds of a single species, such as eelgrass, for the purpose of environmental follow-up. The use of this algorithm with a pixel-oriented classification scheme helped streamline the assessment of interannual differences in eelgrass bed coverage as it is a robust and reproducible mapping tool.

**Fig. 7:**
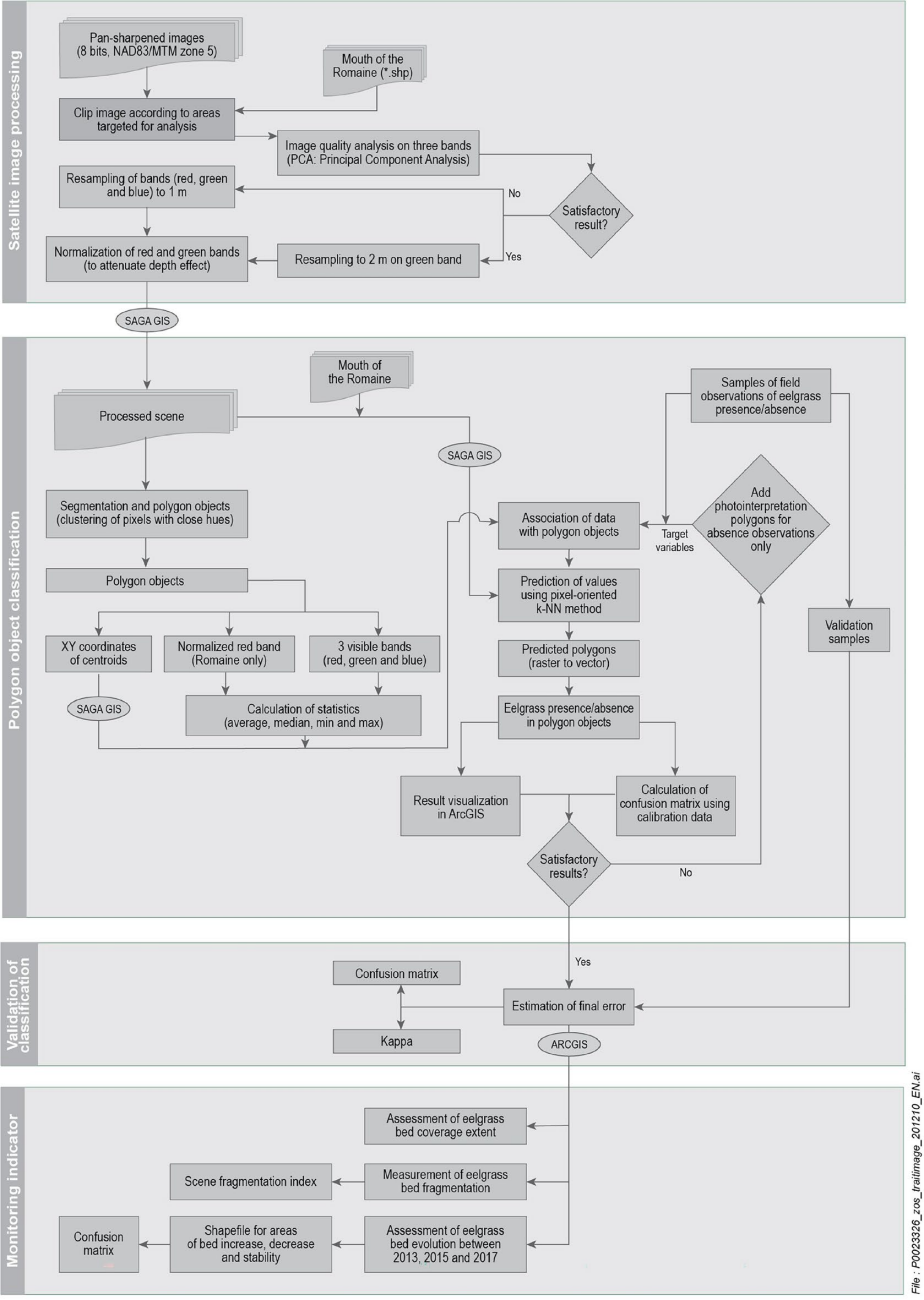
Satellite image processing for eelgrass bed follow-up

The results obtained using two methods for collecting field observations on the presence or absence of eelgrass (transect and random surveying) were subjected to a qualitative assessment. Remote sensing data must be acquired under similar conditions, replicating the field data collection methodology, and similar approaches must be used to implement the pixel-oriented k-NN classification algorithm (Fig. 7). In addition, the mitigation of potential processing errors due to trial-and-error parameterization must be tailored to the zonal statistic requirements of the monitoring area (Table 3) to allow for the reliable assessment of the extent of eelgrass beds over time (Roelfsema et al., 2013).

This study has shown that reducing the offset error between field observations and satellite imagery by resampling that imagery yields training sets that maximize the overlap with remote sensing data. It also demonstrated that the acquisition of high-density field observations does not result in the same pixel-oriented k-NN classification performance as that achieved using the random survey method, where observations are uniformly distributed in the monitoring area.

This paper has shown that applying a consistent methodology with a pixel-oriented k-NN machine language algorithm (Fig. 7) improves eelgrass bed detection and increases the performance significantly, thus helping decrease field-surveying costs, which may be beneficial in remote or hard-to-reach areas.

Future research in this field could benefit from the approach presented in this paper to develop a database to construct a predictive remote sensing model of eelgrass beds. To make accurate predictions, it is important to consider the preprocessing operations and pipelines applied to remotely sensed data prior to providing them as input for the modelling process (Maxwell et al., 2022). Also, other environmental variables must be collected, like water depth, water temperature, salinity levels, turbidity levels and other environmental factors that can impact the growth of eelgrass. If the available data meet these requirements, it may be possible to use advanced AI algorithms to analyze the data and make predictions about where eelgrass is likely to be found, based on empirical local datasets and new satellite imagery. However, it is important to note that predictions made using only past geospatial classified data may not be as accurate as predictions based on past data correlated with some identified environmental factors or water quality factors as Secchi depth and also some climate factors that can change over time and affect the presence of eelgrass. Combining a series of present-absent eelgrass datasets from past years with carefully chosen environmental predictors as detailed above and further model cross-validation with independent historical datasets (e.g. predict two or three existing recent years and check overlapping) can be a roadmap for research development in this area of study.

## Data Availability

The raw data that support the findings of this study are available from the corresponding author, P.L, upon reasonable request.

## References

[CR1] Adams R, Bischof L (1994). Seeded region growing. IEEE Transactions on Pattern Analysis and Machine Intelligence..

[CR2] Alpaydin, E. (2010). Introduction to machine learning. Second edition. MIT Press. Chapter 7 – Clustering, K- mean clustering. pp 7–16

[CR3] Bechtel, B., Ringeler, A., & Böhner, J. (2008). Segmentation for object extraction of trees using MATLAB and SAGA (Version Publisher’s Version). In J. Böhner, T. Blaschke, & L. Montanarella (Eds.), SAGA (Vol. 19, pp. 1–12). *Hamburg: Inst. für Geographie.*

[CR4] Bishop, C. M., & Nasrabadi, N. M. (2006). Pattern recognition and machine learning (Vol. 4, No. 4, p. 738). New York: springer.

[CR5] Bradski, G. (2000). The openCV library. *Dr. Dobb's Journal: Software Tools for the Professional Programmer*, *25*(11), 120-123.

[CR6] CIDCO. (2006). Synthèse critique des outils de télédétection appliquée à la cartographie des habitats benthiques en domaine côtier – Revue bibliographique. Prepared for the Canadian Hydrographic Service (Québec Region) by the Interdisciplinary Centre for the Development of Ocean Mapping (CIDCO). 124 pages and appendices.

[CR7] Demšar U, Harris P, Chris Brunsdon A, Fotheringham S, McLoone S (2013). Principal component analysis on spatial data: An overview. Annals of the Association of American Geographers.

[CR8] DFO. (2009). Does eelgrass (*Zostera marina*) meet the criteria as an ecologically significant species? DFO Canadian Science Advisory Secretariat Science Advisory Report 2009/018.

[CR9] Duarte CM, Chiscano CL (1999). Seagrass biomass and production : A reassessment. Aquatic Botany.

[CR10] EFFIGIS, 2021. https://effigis.com/wp-content/uploads/2017/07/Folio-SATELLITE-IMAGES-earth-in-every-scale.pdf. Accessed 21 Sept 2021

[CR11] Effrosynidis, D., Arampatzis, A., & Sylaios, G. (2018). Seagrass detection in the mediterranean: A supervised learning approach. *Ecological Informatics*,* 48*, 158-170.

[CR12] Franco-Lopez H, Ek AR, Bauer ME (2001). Estimation and mapping of forest stand density, volume, and cover type using the k-nearest neighbours method. Internation Remote Sensing of Environment.

[CR13] Fonseca MS, CahalanJ A (1992). A preliminary evaluation of wave attenuation by four species of seagrass. Estuarine Coastal and Shelf Science.

[CR14] Gollapudi, S. (2019). *Learn computer vision using OpenCV*. Apress.

[CR15] Hemminga, M. A., & Duarte, C. M. (2000). *Seagrass ecology*. Cambridge University Press.

[CR16] Hily, C. & Bouteille, M. (1999). Modifications of the specific diversity and feeding guilds in an intertidal sediment colonized by an eelgrass bed (Zostera marina) (Brittany, France). *C.R. Acad. Sci. Paris, Sciences de la vie*, *322*, 1121–1131.

[CR17] Hossain MS, Bujang JS, Zakaria MH, Hashim M (2015). The application of remote sensing to seagrass ecosystems: An overview and future research prospects. International Journal of Remote Sensing.

[CR18] Production, H. Q. (2007). *Complexe de la Romaine: étude d'impact sur l'environnement.* Hydro-Québec Production.

[CR19] Immitzer M, Atzberger C, Koukal T (2012). Tree species classification with random forest using very high spatial resolution 8-Band WorldView-2 Satellite Data. Remote Sens..

[CR20] Landis JR, Kock GG (1977). The measurement of observer agreement for categorical data. Biometrics.

[CR21] Maxwell AE, Bester MS, Ramezan CA (2022). Enhancing reproducibility and replicability in remote sensing deep learning research and practice. Remote Sensing.

[CR22] McCoy, R. M. (2005). *Field methods in remote sensing. *Guilford Press.

[CR23] OpenCV, I. (2015). Open source computer vision library.

[CR24] Provencher, L., & Deslandes, S. (2012). *Utilisation d'images satellitaires pour évaluer la superficie, l'étendue et la densité de l'herbier de la zostère marine (Zostera marina) de la péninsule de Manicouagan (Québec).* Direction régionale des sciences, Pêches et océans Canada, Institut Maurice-Lamontagne.

[CR25] Ratner, B. (2017).* Statistical and machine-learning data mining:: Techniques for better predictive modeling and analysis of big data. *CRC Press.

[CR26] Roelfsema C, Kovacs EM, Saunders MI, Phinn S, Lyons M, Maxwell P (2013). Challenges of remote sensing for quantifying changes in large complex seagrass environments. Estuarine, Coastal and Shelf Science.

[CR27] SAGA. (2015). Conrad, O., Bechtel, B., Bock, M., Dietrich, H., Fischer, E., Gerlitz, L., Wehberg, J., Wichmann, V., and Böhner, J.: System for Automated Geoscientific Analyses (SAGA) v. 2.1.4, *Geoscientific Model Development*, *8*, 1991–2007. 10.5194/gmd-8-1991-2015

[CR28] Saifi, M. Y., Singla, J., & Nikita. (2020) Deep learning based framework for semantic segmentation of satellite images. *Fourth International Conference on Computing Methodologies And Communication (ICCMC), 2020,* 369–374, 10.1109/ICCMC48092.2020.ICCMC-00069

[CR29] Schweizer D, Armstrong RA, Posada J (2005). Remote sensing characterization of benthic habitats and submerged vegetation biomass in Los Roques Archipelago National Park, Venezuela. International Journal of Remote Sensing.

[CR30] Shapiro, A. C., Trettin, C. C., Kuchly, H., Alavinapanah, S., & Bandeira, S. (2015). The mangroves of the Zambezi Delta: increase in extent observed via satellite from 1994 to 2013. *Remote Sensing,**7*(12), 16504–16518.

[CR31] Vandermeulen, H., Surette, J., Skinner, M., & Department of Fisheries and Oceans, Ottawa, ON(Canada); Canadian Science Advisory Secretariat, Ottawa, ON(Canada). (2012). *Responses of eelgrass (Zostera marina L.) to stress *(No. 2011/095). DFO, Ottawa, ON(Canada).

[CR32] Vandermeulen, H., & Department of Fisheries and Oceans, Ottawa, ON(Canada); Canadian Science Advisory Secretariat, Ottawa, ON(Canada). (2005). *Assessing marine habitat sensitivity: a case study with eelgrass(Zostera marina L.) and kelps(Laminaria, Macrocystis)* (No. 2005/032). DFO, Ottawa, ON(Canada).

[CR33] Vandermeulen, H. (2009). *An introduction to Eelgrass (Zostera marina L.): The persistent ecosystem engineer.* Fisheries and Oceans Canada, Science, Maritimes Region.

[CR34] Wang, L. (2019). Research and implementation of machine learning classifier based on KNN. *IOP Conference Series: Materials Science and Engineering,* 677 052038, 3.

[CR35] Wesolkowski, S., & Fieguth, P. (2001). Color image segmentation using vector angle-based region growing. In *Proc. SPIE 4421, 9th Congress of the International Colour Association*, 910 (6 June 2002). 10.1117/12.464654

